# Tick-, Flea-, and Louse-Borne Diseases of Public Health and Veterinary Significance in Nigeria

**DOI:** 10.3390/tropicalmed3010003

**Published:** 2018-01-03

**Authors:** Oluwaseun Oguntomole, Ugochukwu Nwaeze, Marina E. Eremeeva

**Affiliations:** Jiann-Ping Hsu College of Public Health, Georgia Southern University, 501 Forest Drive, Statesboro, GA 30458-8015, USA; oo00802@georgiasouthern.edu (O.O.); un00078@georgiasouthern.edu (U.N.)

**Keywords:** tick, flea, louse, vector-borne diseases, rickettsial diseases, public health, veterinary health, Nigeria

## Abstract

Mosquito-borne diseases are common high-impact diseases in tropical and subtropical areas. However, other non-mosquito vector-borne pathogens (VBPs) may share their geographic distribution, seasonality, and clinical manifestations, thereby contributing their share to the morbidity and mortality caused by febrile illnesses in these regions. The purpose of this work was to collect and review existing information and identify knowledge gaps about tick, flea-, and louse-borne diseases of veterinary and public health significance in Nigeria. Full-length articles about VBPs were reviewed and relevant information about the vectors, their hosts, geographic distribution, seasonality, and association(s) with human or veterinary diseases was extracted. Specific laboratory tools used for detection and identification of VBPs in Nigeria were also identified. A total of 62 original publications were examined. Substantial information about the prevalence and impacts of ticks and fleas on pet and service dogs (18 articles), and livestock animals (23 articles) were available; however, information about their association with and potential for causing human illnesses was largely absent despite the zoonotic nature of many of these peri-domestic veterinary diseases. Recent publications that employed molecular methods of detection demonstrated the occurrence of several classic (*Ehrlichia canis*, *Rickettsia africae*, *Bartonella* sp.) and emerging human pathogens (*R. aeschlimannii*, *Neoehrlichia mikurensis*) in ticks and fleas. However, information about other pathogens often found in association with ticks (*R. conorii*) and fleas (*R. typhi*, *R. felis*) across the African continent was lacking. Records of louse-borne epidemic typhus in Nigeria date to 1947; however, its current status is not known. This review provides an essential baseline summary of the current knowledge in Nigeria of non-mosquito VBPs, and should stimulate improvements in the surveillance of the veterinary and human diseases they cause in Nigeria. Due to increasing recognition of these diseases in other African countries, veterinary and public health professionals in Nigeria should expand the list of possible diseases considered in patients presenting with fever of unknown etiology.

## 1. Introduction

Vector-borne microorganisms, including numerous pathogens, persist in nature in various arthropods whose prevalence may be expanding due to global warming and human activities that promote favorable conditions for these arthropods. These activities include farming practices, community sports and amusements, globalization of travel and trade, and forest encroachment, which facilitate human exposure to agents propagated in these modified environments [1,2,3,4,5]. They may also ease the transmission of previously rare agents and increase the numbers of infected hosts and carriers, mostly through more frequent exposure of naïve hosts and domestic animals to these vectors.

Mosquito-borne diseases are common high-impact diseases in tropical and subtropical areas and receive most of the arthropod-directed attention from public health professionals. However, other vector-borne diseases and their pathogens (VBPs) may share their geographic distribution, seasonality, and clinical manifestations, thereby contributing their share to the morbidity and mortality caused by febrile illnesses in this region. Rickettsial diseases and their pathogens are one such largely silent contribution, as has been previously shown in the context of the overlapping epidemiology of malaria and infections caused by *Rickettsia felis* in sub-Saharan Africa [6]. Several other reviews and research articles demonstrated the broad spread and distribution of rickettsial pathogens in many regions on this continent [7,8,9]. However, in Nigeria, while there are numerous examples of veterinary research focused on tick-borne diseases in livestock from 1923 to 1966 and from 1966 to 1976 [10,11], recent updates from this area are largely missing.

**Figure 1:**
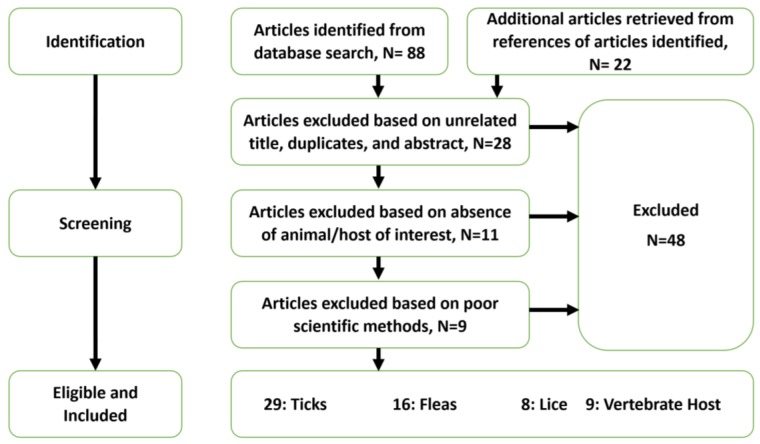
Flow diagram for the selection of articles for review.

The purpose of this work was to conduct an in-depth review of the historic and recent studies in Nigeria that focused on ticks, fleas, lice, and the pathogens they transmit. Full-length articles published from 1940 to the present were identified through PubMed, African Journals Online, and Google Scholar searches, and additional publications were retrieved by reviewing the references cited in every article identified (Figure 1). Articles without records of collection locations of ticks, fleas, lice, or the pathogens and diseases they transmit in Nigeria, were excluded. Also, articles without identifying the vector host of concern were excluded. Sixty-two peer-reviewed articles were identified. All articles were reviewed and relevant information about the vectors (ticks, fleas, and human lice), their hosts, geographic distribution, seasonality, and association(s) with human or veterinary diseases, was recorded. Laboratory tools used for detection and identification of VBPs were also examined.

### Nigeria: Demographics, Geography, Climate, Ecology, and Practices

**Figure 2:**
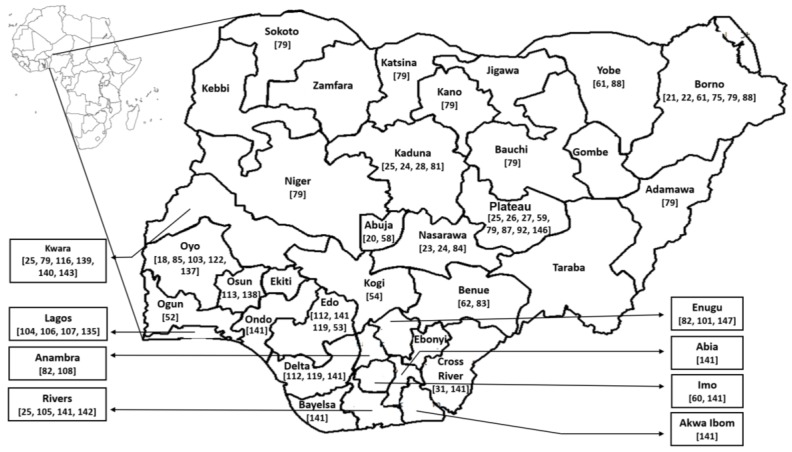
Geographic location of Nigeria and identification of major areas where vector-borne diseases were studied. Numbers indicate references reporting vector-borne diseases in the corresponding state.

Nigeria is located on the west coast of Africa. It shares its boundary to the north with Niger, to the west with the Republic of Benin, and to the east with Cameroun and Chad (Figure 2).

Nigeria is only the 14th largest country in Africa (of 57), but it is the most populous with over 170 million people and more than 250 ethnic groups. The country spans about 923,768 sq. km. (356,668.67 sq. miles) in land area with a population density of 171.32 persons per sq. km (443.71 persons per sq. mile) [12]. Its geographical terrain ranges from high savannah-covered plateaus in the north, rainforests inland and swamps in the southern coast, and the oil-rich Niger Delta in the south. The climatic conditions differ significantly among major regions, with variable wet and dry seasons. There is a gradual decrease in the average yearly rainfall from the south to the north of the country ranging from 3000 mm a year in the southeast, 1800 mm a year in the southwest, to 500 mm a year in the far north [13]. The maximum mean temperature in the north is higher than in other regions, while the minimum mean temperature in the northern region is lower with high humidity for most part of the year [13]. In the southern coast and Niger Delta region, maximum temperatures seldom rise above 32 °C while the minimum temperature during the wet season is usually about 22 °C. In the northern region, especially northeastern and north–central region, the temperature can rise above 38 °C, while frost may appear during the night in the mountainous areas in the same season with temperatures dropping to about 12 °C in this part of the country [14,15].

There are various structures, establishments, and cultural practices like wildlife reserves, museums, and coastal beaches which attract tourists to the country with more than 2 million people visiting annually in the early 2000s [13]. The number of people visiting these historical structures and centers has significantly declined since 1999 due to recurrent periods of ethnic violence and the menace of the Nigerian Islamist Movement popularly known as ‘Boko Haram’, especially in the northern region of the country [16]. This political situation has led to the displacement of more than 2.8 million people, disruption of farming and animal rearing, hindered development in the region, and is of great concern as it could ultimately lead to uncontrolled propagation of ectoparasites on domestic stock and spread of their associated pathogens.

Below we summarize individual vector groups, the current knowledge base about ticks, fleas, human lice, and the pathogens they transmit in Nigeria, discuss their impact on human and veterinary health in the region, and outline future directions for the study and prevention of these diseases and approaches for control of the major vectors implicated in their transmission.

## 2. Ticks in Nigeria

There is a long history of studying ticks and tick-borne diseases in Nigeria mainly due to their significant impact on peri-domestic animals, including herds and service dogs. Classic studies were conducted to determine the distribution of ticks and tick-borne pathogens across the country; they relied on the morphological identification of ectoparasites and detection of pathogens through examination of hemolymph smears and other laboratory staining techniques [17,18,19,20,21,22,23]. The most recent studies included serological and molecular techniques [24,25,26,27,28,29].

### 2.1. Summary of Tick Species Known to Be Present in Nigeria

The Ixodid ticks of four hard tick genera—including *Amblyomma*, *Rhipicephalus*, *Hyalomma*, and *Haemaphysalis*—are commonly known and collected from animals in Nigeria (Table 1). Furthermore, the argasid soft tick, *Ornithodoros moubata*, is known to attack humans and transmits etiologic agents causing human relapsing fever and African swine fever [30]. As in other tropical countries, the brown dog tick, *Rhipicephalus sanguineus*, is the most reported tick in Nigeria [30]. This is mostly due to its high feeding preference for domestic dogs and its ability to survive on other vertebrate hosts [30]. It has been reported to occur in all regions in the country but most frequently in the southern regions of the country in contrast to the northern region where *Rhipicephalus* (*Boophilus*) *decoloratus* and *Amblyomma variegatum* occur more frequently.

Climatic factors have a significant impact on the natural development of ticks in Nigeria; they influence tick oviposition and larval hatching patterns for each species, duration of the various developmental periods, and the progression of their developmental stages [31,32,33,34,35,36,37,38,39,40]. Most of these studies focused on the oviposition and hatching pattern of varied species of ticks in natural conditions [33], in laboratory settings [31,32,35,37,38], or both [34]. In a study conducted by Dipeolu [33], the developmental periods of *A. variegatum*, *Hyalomma rufipes*, *Hyalomma truncatum*, *Rhipicepahlus geigyi*, and *Rhipicephalus decoloratus* collected from slaughter cattle in Ibadan (Oyo State, southwest coastal Nigeria) were recorded from egg to adult stages under natural conditions for a year. There was no variation in the attachment and engorgement periods of each tick species. Eggs of all the ticks except *Rh. geigyi* hatched faster in the dry season than the wet season. Eggs of *A. variegatum* hatched faster with a mean difference ranging from 2 to 18 days, while eggs of *B. decoloratus* had a mean hatch time difference ranging from 2 to 11 days [33]. The molting periods were longer during the wet season than the dry seasons for all the ticks [33]; *A. variegatum* molted for 2–13 days longer during the wet season than the dry season [33]. Overall, the ticks thrived better in the open areas under all observed natural conditions except for *Rh. geigyi* which preferred shaded areas [33]. Furthermore, at a certain time during the year the development of the tick eggs of some species halted or decelerated: *A. variegatum* did not hatch in December and January, *Rh. Geigyi*, did not hatch from December to February, while *H. truncatum* failed to hatch from August to October. The duration of the life cycle of *A. variegatum* increased from 41 to 65 days during the dry season to 44–77 days at the beginning of the rainy season, and to 77–116 days at the peak of the rainy season [33]. Similar changes were recorded in other tick species studied except *Rh. geigyi* where a slight reduction in the duration of the life cycle was observed. Therefore, it is believed that alterations in the duration of the life cycle affect the prevalence of these ticks and their geographic and seasonal variations in Nigeria [33]. The presence of *Rh. geigyi* has been documented in the savannah and forest zones but not in the hot and dry Sahel zones of the northern region, while *H. rufipes* is established in the wet and humid southern region [33]. Similar observations were obtained in studies where the natural conditions were reproduced in the laboratory to observe varied tick species [31,35]. The ticks were observed to lay more eggs when fully engorged; oviposition and eclosion periods were shorter at high temperatures (≥24 °C) [31]. At 15 °C, all the eggs, except those of *H. rufipes*, did not hatch, suggesting that *H. rufipes* is the most tolerant of these species [31]. Lastly, the duration of oviposition and the size and number of eggs laid were highest among same tick species collected from cattle, followed by those from sheep and then horses [35].

Some of the traditional control measures employed locally by livestock farmers in Nigeria include: maintaining a hygienic breeding area, manual removal of ticks, use of herbs and incantations, bush burning, and movement of animals to other breeding sites [27,39,65,66]. The contemporary control measures and surveillance system adopted in Nigeria are based on the Integrated Disease Surveillance and Response (IDSR) program—a strategy developed by the WHO in 2000 to improve historically poor surveillance systems in the African sub-region [67]. This surveillance system is still limited and weak, mostly due to the paucity of the qualified public health workforce needed to operate the system [68,69]. To fill this gap, a public health competency-based training program was initiated, in conjunction with the US CDC, in 2007 [70]. This program was established to foster and implement a feasible, effective, and adequately staffed multi-disease public health surveillance system—including zoonotic diseases—in all states and local government areas in the country by 2020 [70]. There was little or no support from the government through legislation or funding to control these ticks in the past [65], although these recent efforts were undertaken to improve the situation [70]. Nevertheless, ticks remain a critical focus of concern in regard to public health safety in Nigeria.

### 2.2. Impacts of Ticks and Tick-Borne Diseases on Dogs and Cats

People own dogs and cats in Nigeria for various reasons based on their social status, culture, and occupation [25,58,71,72]. Dogs raised and retained in residential areas are primarily used for herding cattle, especially in northern regions, and for security purposes. Most of these dogs are not kept in strict confinement but are often allowed to roam and stray. Due to these practices, the dogs are exposed to a variety of potential health hazards such as infections from parasites and their vectors, while simultaneously serving as reservoirs for potential transmission of infectious diseases to humans. In addition to these purposes for owning dogs, they are also used as a food source among some ethnic groups in Nigeria [25,71]. There are abundant but poorly documented populations of owned and stray dogs and cats with little effort towards controlling their numbers. In an early pilot study, it was determined that the dog to human ratio was 1:21 for urban areas and 1:45 for rural areas suggesting a greater population of dogs exists in urban areas of Nigeria [72]. These estimates are low compared to what is recorded in other regions like the USA, Mexico, and some European countries [73,74]; however, no recent studies or census numbers have been found for assessing the current population of pets in Nigeria.

The brown dog tick, *Rh. sanguineus*, is the most prevalent tick found on dogs in Nigeria [21,40,47,55,58]. Its prevalence varies both geographically and seasonally in Nigeria. In a recent study, the prevalence of *Rh. sanguineus* infestation in 200 dogs was reported to be 80% in Lokoja, Kogi State (south-central Nigeria) compared to only about 10.8% in Borno, in the northeastern part of Nigeria where the *Rhipicephalus* (*Boophilus*) cattle ticks are more prevalent [21,45]. Most of the dogs examined in Kogi state were used as pets, house-guards, or for hunting, while all the dogs examined in Borno state were stray dogs. Assessing the cumulative pattern, it was observed that ticks were more prevalent during the rainy season in Borno State [21], which contrasts with the findings from an older study conducted by Dipeolu [33] in Oyo State.

Similar to other parts of the world [75,76], Nigerian *Rh. sanguineus* preferentially feeds on dogs but will also bite humans and other animals; it is also capable of house infestations. In a report about a heavy house infestation in Lagos [40], *Rh. sanguineus* was found on three dogs, the outside walls of the house, and in the garage where the dogs were kept at night. These observations indicate a potential for establishing an endemic focus, which may lead to an outbreak of diseases in a scenario with little awareness about associated pathogens transmitted by these ticks as has been previously described in other countries [75].

Several recent investigations focused on detection of various pathogens found in association with dogs and ticks collected on dogs [20,21,22,24,25,28,61,62,77]. Most of the studies except one were conducted in the central eastern region especially in the Plateau State [20,24,25,28,61]. Three species of ticks were predominantly collected, including *Rh. sanguineus* with an infestation rate ranging from 0.3 to 80%, *A. variegatum* (0.3–70.2%) and *H. leachi* (4.4–33.2%) in different studies [21,24,25,28,45,47,48,54,57]. DNA of *Babesia* spp., *Anaplasma* spp., and *Rickettsia* spp. were detected most frequently [20,21,24,25,28,61].

Kamani et al. [25] tested 181 blood samples and 258 ticks (*Rh. sanguineus*, *Rh. turanicus*, and *Haemaphysalis leachi*) collected from 42 dogs in 4 states (Plateau: east-central; Kaduna: north-central; Kwara: west-central; and Rivers: mid-east-coastal). DNA of *Hepatozoon canis* was the most prevalent (41.4%) in the blood samples, while DNA of *Ehrlichia canis* (23.7%) was the most prevalent among the 76 tick pools tested. DNA of *Anaplasma platys*, another canine pathogen was detected for the first time in Nigeria in 6.6% of canine bloods and one tick pool of *Rh. sanguineus*. DNA of *Rickettsia* spp. was detected using *glt*A PCR in 6.6% of canine bloods and 10.5% of tick pools; DNA of *Rickettsia conorii israelensis* was detected in one dog and one *Rh. sanguineus* pool DNA while DNA of *Candidatus* Neoehrlichia mikurensis was detected in four (5.3%) of the tick pools tested, including two *Rh. sanguineus*, and one each of *Rh. turanicus* and *H. adleri*. This was the first detection of *Candidatus* N. mikurensis in Nigeria. This agent is an emerging pathogen affecting humans and animals in Europe and Asia where it has been detected in association with *Ixodes* spp. ticks, rats, and dogs [78,79,80]. Whether the PCR positive ticks had acquired *Candidatus* N. mikurensis from infected animals is unknown since only engorged ticks were tested. Physicians should consider this pathogen to rule out complicated unexplained fever of unknown etiology in Nigerian patients. Furthermore, a role of co-infections is not excluded, since nine (11.8%) of the tick pools tested positive for two or more pathogens [25].

Application of PCR and reverse line blot (RLB) assays to screen for DNA of tick-borne pathogens in canine blood samples from Jos, Plateau State, corroborated previous findings and expanded the list of veterinary pathogens that have been confirmed to be present in the region using molecular techniques [28]. The dogs were predominantly infested by *Rh. sanguineus* (73% of all ticks collected) and *H*. *leachi* (18%); while six other tick species were collected on those dogs, no testing was reported. Based on the RLB testing, 72% of the canine blood samples (*N* = 100) tested positive for one or more pathogen [28]. DNA for seven pathogens was detected including *Babesia rossi* (53%), *Theileria* spp. (13%), *E. canis* (7%), *Anaplasma* spp. (7%), *T. equi* (4%), *B. vogeli* (1%), and *Ehrlichia ruminantium* (1%); evidence of co-infections was obtained from 23 dogs. In comparison, Ogo et al. [24] applied PCR for detection of rickettsial pathogens and RLB to test for *Babesia* spp. and *Theileria* sp. in partially engorged *A. variegatum* and *Rh. decoloratus* from cattle and *Rh. sanguineus* from dogs from three areas in Plateau and Nasarawa states (mid-east central region). In total, 15.1% of ticks tested positive by either method. *Babesia* spp. was only detected in 11% of 153 *A. variegatum* including DNA of *B. bigemina* (1.3%) and *B. divergens* (0.6%), while DNA of *Theileria* spp. was not found. DNA of *R. africae* was detected in all tick species collected in this study including 7.8% of *A. variegatum*, 4.4% *Rh. decoloratus* and 5% *Rh. sanguineus*; *Ehrlichia* spp. including *E. ruminantium* were not detected at all; nine *Rh. decoloratus* tested PCR positive for DNA of *A. marginale*. These contrasting results may be attributed to the differences in the source of ticks collected and/or variable sensitivity of the detection methods used.

Exposure to *R. africae* is usually associated with cattle [24,81], or is reported by tourists after walking through grasslands and being exposed to questing ticks [82]. However, finding *R. africae* in *A. variegatum* from a dog in Plateau State [28] suggests that *R. africae* may be disseminated to human domiciles on dogs even if they are not susceptible to infection. PCR detection of *R. africae* in *A. variegatum* collected from a dog was previously reported from Senegal, a West African country [83]. Furthermore, previous work conducted in the southern district of the Kruger National Park in South Africa reported wild dog infestations with numerous ticks including *A. hebraeum*, and detection of antibodies to *R. conorii* and/or *R. africae* in 27 of 29 dogs tested by indirect fluorescent antibody technique, suggestive of their possible exposure to this or related spotted fever group rickettsiae [84].

Only one study was found that mentions tick-borne diseases of cats in Nigeria [11]. *Hepatozoon felis* was detected in the polymorphonuclear leucocytes of a cat that was part of a summary survey of tick-borne diseases in domestic animals in northern Nigeria during 1966–1976 [11]. It is likely that this pathogen is prevalent in Nigerian felids as has been reported from other African countries, and introduction of molecular methods will substantially improve its detection and identification in this part of the continent [85,86,87].

### 2.3. Ticks and Tick-Borne Diseases in Large Livestock Animals

In Nigeria, ticks and tick-transmitted pathogens represent a major constraint to livestock health and productivity [88]. The most reliable census on livestock populations in Nigeria was conducted in 1990 through a World Bank assisted government project that estimated that there were 13.9 million cattle and 90% of them were in the northern two-thirds of the country [42,89]. Livestock production is a significant contributor to the Nigerian economy. Based on the 2016 estimation by the Ministry of Agriculture and Rural Development, livestock production contributes 20–25% of agricultural gross domestic product (GDP) and 6–8% of the total GDP [90]. About 70% of the Nigerian population is engaged in agricultural practices either for domestic or commercial purpose [90,91]. To evaluate the economic impact of *Babesia bigemina*, an etiological agent of bovine babesiosis (also called tick fever or cattle fever), on cattle productivity, six Zebu cattle were left to graze on a *Rhipicephalus* (*Boophilus* sp.)-infested pasture at the Ministry of Agriculture demonstration farms in Ibadan where ticks infected with *B. bigemina* were present [17]. Following the infection, an average lean mass weight loss of 9 kg in these cattle was observed and a significant estimated average annual loss of 360 million Naira was predicted. The error in this small study cannot be estimated but may even be underestimated since cattle are consistently involved in extensive grazing in forest and pasture, and exposed to ticks, especially in the wet season occurring from April to October, but less in the dry season, from November to March [27]. While there are no indigenous camels in Nigeria, one-humped camels (*Camelus dromedarius*) are often imported from Chad, Mali, Libya, and Niger, mainly for transportation and meat purposes. The estimated population of camels in northern Nigeria was 90,000 in 1990 and 74,360 in 1992 [89,92] but no census on the current population of these animals in Nigeria could be found.

Several articles evaluated the extent of tick infestation of livestock animals in Nigeria [23,42,44,46,48,56,60,93]. *Hyalomma* sp., *Rhipicephalus* (*Boophilus* sp.), *A. variegatum*, and *Rh. sanguineus* were the most common ticks identified in these studies. Other tick species—including *H. leachii*, *Ornithodoros* sp., and *Amblyomma lepidum*—were found to infect livestock animals less frequently [23,42,48]. The earliest study of tick infestation of cattle in Nigeria was conducted by Unsworth [60] to determine the distribution of the species of ticks that infest cattle in Nigeria. Ticks were collected monthly from cattle in their rearing areas in the north for a year. There were 17 species of ticks collected over this period but *A. variegatum* and *Rh. decoloratus* were the most common and widely-distributed species collected [60]. In Borno State (Northeast most) and adjacent Yobe State, 2200 cattle, and 1600 camels were examined for ectoparasites [48]. Among 3620 ticks collected from the camels, *Hyalomma rufipes* (34.86%), *H. dromedarii* (30.38%), *Rh. decoloratus* (24.20%), and *H. truncatum* (10.55%) were identified. 81.8% of the cattle were infested; *Rh. decoloratus* (21.78%), *H. truncatum* (17.9%), *Rh. evertsi* (17.84%), *H. rufipes* (11.46%), *A. variegatum* (11.37%), *Rh. sanguineus* (8.9%), *H. leachii* (8.77%), and *A. lepidum* (1.97%) were identified among the 5391 ticks collected. The cattle were infested by the same ticks collected from camels except *H. dromedarii* exhibited host specificity. Similar observations were recorded when tick infestations of cattle and camels were investigated in Oyo and Nasarawa states [50,51].

We have identified only one study where questing and feeding ticks collected from cattle from the same collection site were tested simultaneously [81]. Accordingly, 700 questing ticks were collected by cloth dragging and direct hand-picking from vegetation at 7 locations and 136 feeding ticks from 11 herds of cattle from 4 locations in Oyo State in southwestern Nigeria [81]. Questing ticks from the vegetation were all *Rh. evertsi* while *Rh. annulatus* (37.5%), *A. variegatum* (33.8%), *H. impeltatum* (14.7%), and *Rh. Evertsi*, (14%) were collected from the cattle. Feeding ticks tested PCR positive for DNA of *Anaplasma* spp. (11%), *Rickettsia* spp. (12.5%), *Theileria mutans* (2.9%), and *Coxiella burnetii* (14%). DNA of *Anaplasmataceae* was detected in all four feeding tick species tested; *Anaplasma marginale* and *A. centrale* were the most prevalent (53.3%; 8/15); DNA of both *E. chaffeensis* and *E*. *ewingii* was detected only in a single tick, and unidentified *Ehrlichia* spp. were detected in five ticks. DNA of *R. africae*-like species was commonly detected in all four species of feeding ticks (82.4%; 14/17) with *R*. *aeschlimannii* being the second most predominant *Rickettsia* species (17.6%; 3/17). Based on these findings, it was estimated that 45.5% (5/11) of herds were infected with *A. marginale*/*centrale*, 45.5% (5/11) with *Ehrlichia* sp., 9.1% (1/11) with *E. chaffeensis*, and 9.1% (1/11) *with E. ewingii*. The estimated infection rate of cattle in *Rickettsia*-positive herds ranged from 15.4 to 50% for *R. africae* and 7.7 to 100% with *R. aeschlimannii*, although these numbers must be interpreted with caution since only a single tick or/and animal tested positive in these herds. PCR positivity of questing ticks was lower and only DNAs from *Rickettsia* spp. (3.1%), *C. burnetii* (0.1%), and *Borrelia* spp. (0.4%) were detected. Sequencing of a 339-nt fragment of the 17-kDa protein gene of rickettsiae permitted identification of *R. massiliae* in *R. evertsi* questing ticks from all seven locations of tick collections [81]. It was also reported that DNA of a *Rickettsia* sp. belonging to the *R. rickettsii* group was detected in one of the ticks; however, it is likely a different *Rickettsia* sp., since the 17 kDa protein gene does not allow accurate species identification due to its low nucleotide diversity [94]. DNA from *C. burnetii* was detected both in ticks collected from cattle and the vegetation; at least one tick of each species was PCR positive [81]. In total, 63.6% (*N* = 11) of herds were infested with ticks positive for *Coxiella* and in most cases (57.1%, *N* = 7), more than one animal per herd was involved.

*Amblyomma variegatum* is prevalent in the North central part of Nigeria, which includes Plateau, Kaduna, Kwara, Niger, Nasarawa, and Benue States, and the Federal Capital Territory [27]. These areas are the major cattle-rearing centers. In Plateau State, this tick was common on cattle [24,27,41]. Ticks were also collected from Zebu cattle imported from Uganda [41]. DNA of SFG *Rickettsia* was detected by PCR in 62% of *A. variegatum* (141 ticks) collected from cattle in Plateau State. *Amblyomma variegatum* was also collected in some studies conducted in other states (Oyo: east-central; Nasarawa: central, and Borno and Yobe in the northeast) [48,49,50,51]. The highest tick infestation rate of 43.8% was recorded in Borno and Yobe state by Opara et al. [49] when 3150 randomly selected cattle were examined for ticks by manual handpicking.

Because of their veterinary and economic impacts on cattle, assessments of the circulation of two *Anaplasmataceae*, *E. ruminantium*, and *A. marginale*/*centrale*, in ticks are very important for Nigeria, as in many other African countries. *Ehrlichia ruminantium* causes heartwater (cowdriosis). The presence of this pathogen has been reported in Nigeria [63,95,96,97,98,99,100]. It was first briefly discussed in two consecutive systematic reviews summarizing veterinary research conducted in 1923–1966 and 1966–1976 [10,11]. Heartwater is endemic in northern Nigeria [96,100]; since it affects cattle, goats and sheep, strains of *E. ruminantium* with different tropisms and genetic make-up may exist in Nigeria as in other African countries [101,102].

Lorusso et al. [63] determined the occurrence of tick-borne pathogens in cattle in Plateau State using PCR-based reverse line blotting and sequencing. 82.6% of the 774 cattle blood samples had DNA from at least one pathogen [63]. However, DNA of *E. ruminantium* was detected in only eight (1.1%) of the blood samples using the 460–520-bp long fragment from the V1 hypervariable region of the 16S rRNA gene for *Ehrlichia* spp. [63]. The authors attributed the low prevalence of *E. ruminantium* to the biology of its infection (low reproduction of microorganism in the endothelial cells of the capillaries and infrequent release into a bloodstream [63]. On the other hand, *Anaplasma* sp. (Omatjenne) was another frequently detected microorganism in this study at a prevalence of 34.7%. This bacterium was originally isolated from *H. truncatum* in Namibia; after passage through three generations in *A. hebraeum*, it became infectious when experimentally inoculated in sheep and caused a disease indistinguishable from heartwater [103]; the Omatjenne genotype has also been detected in ticks collected from Nigerian dogs in the same area as the study by Lorusso et al. [28,63]. *A. marginale* (39.1%), *A. centrale* (6.3%), *A. platys* (3.9%), *R. massiliae* (3.5%), and *B. bovis* (2.0%) were also detected; however, *Theileria* spp. (39.5–66.3%) were the most prevalent microorganisms detected. The most critical finding of the study was that 69.6% of cattle were positive for two or more microorganisms simultaneously; 77 of various combinations of microorganisms were diagnosed [63].

*Anaplasma marginale* is the most prevalent tick-borne livestock pathogen in Nigeria. It affects economic production of cattle because it causes significant loss of milk production and weight, and it often leads to death of the infected cattle [104]. Control measures are important to prevent infection because once infected, cattle are usually carriers for life [104]. *Anaplasma marginale* has been detected historically in livestock animals in Nigeria through examination of blood smears [10,105,106]. Subsequently, serological methods were adopted to detect this pathogen [24,64]. In the study conducted by Ajayi & Dipeolu [64] in 10 northern states, serum samples were collected randomly from 500 cattle to assess the presence of *A. marginale*, *Babesia bigemina*, and *B. bovis* using multiple serological tests. 79.4% of the serum samples were positive for *A. marginale* by indirect fluorescent antibody test, 40% by card agglutination test, and 25% by capillary tube-agglutination test [59].

Camels may be essential vertebrate hosts for maintaining ticks and associated tick-borne pathogens as suggested by finding DNA of *R. aeshlimannii* in 43.3% (42/97) pools of 197 *Hyalomma* sp. ticks collected off 51 *C. dromedarius* in Kano, northern Nigeria [29]. Furthermore, the *glt*A fragment of *Rickettsia* spp. was also detected by PCR in 18.8% (*N* = 170) of camel blood samples tested. Unfortunately, their sequence identification was not completed, but it is possible camels are the reservoirs for *R*. *aeschlimannii*, a known agent of spotted fever rickettsiosis in humans [7].

### 2.4. Ticks and Tick-Borne Diseases in Other Livestock Animals

There were 34.5 million goats, 22.1 million sheep, and 3.5 million pigs in Nigeria in 2009 [89]. Few studies in Nigeria have focused on tick infestation of these livestock animals [23,44,48,59]. James-Rugu and Jidayi [48] reported that 26.5% of 400 goats and 43% of 500 sheep were infested by ticks, while no ticks were identified on pigs in four selected local government areas in Borno and Yobe States in Northeast Nigeria. *A. variegatum* and *Rh. decoloratus* were collected from the sheep; however, information about tick species collected from the goats was not reported [48]. In a 2016 study conducted in Nasarawa state [23], 72 and 32 blood samples were collected from goats and sheep, respectively, at an abattoir and examined for the presence of haemoparasites through microscopic examination of blood smears. *Anaplasma* (13.5%), *Babesia* (4.8%) and *Trypanosoma* (1.9%) were detected in blood from 21 animals including 23.61% of the goats and 12.5% of the sheep; however, species identification of these three pathogens was not reported.

### 2.5. Ticks and Tick-Borne Diseases in Humans

Despite the increasing incidence and prevalence of tick-borne diseases in humans in many developing countries, similar information from Nigeria has not been reported [107]. An outbreak of dermatitis was reported in 1973 among school children and soldiers in Nsukka, Enugu State [108]. Larvae of *A. variegatum* that were found in great numbers on grass blades within the school compound were implicated as the source of the outbreak. Although it was not recognized at the time, it cannot be excluded that the cases were due to infection with *R. africae*. *Rickettsia africae* causes African tick-bite fever, which presents with ‘flu-like symptoms, a characteristic rash and one or more eschars arising from infected larval tick bites [7].

Several cases of *Rh. sanguineus* infestation of humans and animals in 2001 were reported from Owerri, Imo State (southeast coastal region) [57] (11 human, 2 canine, and 3 sheep infestations). Multi-host parasitism was believed to be due to extensive bush clearing in the area resulting in non-availability of alternative animal hosts. The presence of the pathogens causing African tick-bite fever, babesiosis, and tick typhus have been well documented in Nigeria [24,25,41,77,81,93]; however, the diagnosis of these diseases in humans is rarely reported mostly because their primary symptoms are similar to other highly endemic diseases in Nigeria such as malaria and typhoid fever and confirmatory laboratory testing is rare.

## 3. Fleas in Nigeria

In comparison to studies on ticks in Nigeria, more studies and reports on flea infestation of humans as well as their role in the transmission of pathogens were found [109,110,111,112,113]. This could be because flea infestation causes more profound and unpleasant acute symptoms than tick infestation including intense skin itching and pain, generalized rash, and severe allergic reactions. This makes them important to clinicians and laboratory researchers. Several species of fleas have been identified on humans and domesticated animals in Nigeria including the human flea (*Pulex irritans*), dog flea (*Ctenocephalides canis*), jigger flea (*Tunga penetrans*), Oriental rat flea (*Xenopsylla cheopis*) and cat flea (*Ctenocephalides felis*) (Table 2).

*Tunga penetrans* (chigoe flea or jigger) is usually found in human dwellings but it commonly also infests dogs, cows and pigs, as well as humans, in Nigeria [111]. *Echidnophaga gallinacea*, commonly known as the hen flea, stickfast flea, or sticktight flea, was collected from dogs in a study conducted in Anambra State (east central coastal region) [114]. This flea is known to infest other animals and humans but does not transmit any known disease [115]. One study reported its presence on dogs in Nigeria [114]. In this study, 338 dogs, including pets and hunting dogs, were brought to veterinary clinics in Enugu State (south central region); *E. gallinacea* was collected from seven of the dogs [114].

### 3.1. Fleas and Flea-Borne Pathogens in Rats

Knowledge regarding the population sizes and distribution of rat species in Nigeria is limited, but these rodents are known to live in close proximity to human dwellings especially in overcrowded communities and unhygienic environments [26,116]. The Oriental rat flea, *Xenopsylla cheopis*, has only been detected on *Rattus rattus* and *R. norvegicus* in Nigeria [26,43,117,118].

*Rattus rattus*, *R. norvegicus*, and *Cricetomys gambianus* were suggested to be a part of the natural cycle of several *Bartonella* spp. [26]. Accordingly, 32 ectoparasites were collected from 177 rodents in Plateau State, Nigeria. Blood samples from the rodents and the ectoparasite samples (*Ctenophthalmus* spp., *X. cheopsis*) were analyzed using PCR for *Bartonella* spp. DNA of *Bartonella* was detected 26% of blood samples from rodents and 28% of ectoparasite samples. Among the *Bartonella* sequences derived from *R. norvegicus*, 26 were 98–100% similar to *B. elizabethae*, 9 were 97–98% similar to *B. tribicorum*, and 1 was 98% similar to *B. grahamii* [26]. The majority of *Bartonella* DNA detected in blood of *R. rattus* and all the sequences from *C. gambianus* exhibited 98–100% similarity to the homologous sequences of *B. elizabethae* [26]. These pathogens are associated with clinical diseases of humans [119]; however, further work is needed to understand the details of their ecology and the epidemiology of human exposure in the country.

Murine typhus is a zoonotic disease caused by the bacterium, *Rickettsia typhi*, which is most commonly transmitted by *X. cheopsis.* The seroprevalence of *R. typhi* detected among African countries, like Ivory Coast and Mali, varied from 1 to 24% [120], and 34% in Madagascar [121]. Although no reports regarding the occurrence of murine typhus in Nigeria were found, the potential for the transfer of this disease from neighboring countries, especially by shipping, should not be neglected due to the cosmopolitan nature of its vector and rat host. Although *C. felis* is common in Nigeria, it is not known if it also transmits *R. typhi* as it has been implicated in other regions [122,123].

### 3.2. Fleas and Flea-Borne Pathogens in Dogs and Cats

Dogs and cats are usually infested by common species of fleas which transmit flea-borne diseases to humans. In Nigeria, *C. canis*, *P. irritans*, *E. gallinacea*, and *T. penetrans* are most commonly found on dogs and cats [44,53,114]. The cat flea, *Ctenocephalides felis*, is closely related to *C. canis* and it is believed to be more common on dogs than *C. canis* globally [124,125]. However, different observations were obtained in Kwara State (West Central), Nigeria [53]. Dog fleas, *C. canis* (32.1%) were the most prevalent, flea detected on 155 of 396 dogs followed by *P. irritans* (6.6%) and *T. penetrans* (0.5%). Similarly, *C. canis* was also the most prevalent flea detected on dogs in the southeastern region (Edo, Delta, Anambra) of Nigeria [52,114]. *Echidnophaga gallinacea* was detected on 2.1% of the 338 dogs examined by Chukwu [124]. No Nigerian studies were found which focused on flea-borne pathogens in canine and feline blood. The impact of these pathogens is probably significant given the levels of flea infestation that occur, but zoonotic peridomestic transmission of flea-borne pathogens is largely overlooked in Nigeria.

### 3.3. Fleas and Flea-Borne Pathogens in Livestock

Fleas parasitizing dogs and cats can also infest livestock animals and be a major nuisance in livestock operations. Several flea species—including *C. felis*, *C. canis*, *P. irritans*, *T. penetrans*, and *X. cheopsis*—can breed in poultry houses and other livestock farming operations, especially within bedding, straw, sawdust, and sand where these animals are housed [48,107,129]. Consequently, the animals, workers, and owners of these farms can also get infested. Most of the studies conducted on fleas affecting livestock in Nigeria were focused on determining the prevalence of infestation (Table 2).

Heavy infestation of *C. canis* in goats and sheep in Nigeria has been reported [44,107,126,130]. One case report documented infestation of 7 out of 25 Fresian calves on a dairy farm in Nigeria [128]. *Ctenocephalides felis* was identified as the cause of the infestation based on examination of ectoparasites and skin scrapings examined at the parasitology laboratory in Plateau State [128]. Although blood samples were collected to screen for haemoparasites, no infections were reported. In a study by *Tongjura* et al. [50], 1200 cattle, 1200 sheep, and 1200 goats were examined for common ectoparasites of livestock in Nasarawa State (central region); the prevalence of flea infestation was highest among cattle (50.7%) followed by sheep (41.8%), and goats (32.6%); unfortunately, the species of fleas collected were not reported.

### 3.4. Fleas and Flea-Borne Pathogens in Humans

Only *P. irritans* is typically referred to as a human flea; however, other fleas may come in contact with humans through interaction with dogs, cats, and livestock animals and transmit zoonotic pathogens. Similarly, *P. irritans* is often seen on dogs, cats, rats, goats and other livestock animals, so it actually has a cosmopolitan distribution [124].

Fagbemi et al. [109] described a massive infestation of humans by *C. canis* in a household surrounded by lawns and shrubs which occurred after a large scale elimination of stray dogs in the area. In contrast, reports of human exposure to *C. felis* in Nigeria were not found, despite the cosmopolitan nature of this flea. In other countries across Africa there are numerous reports of exposure to cat flea-borne *R. felis* and increased recognition of cases of cat flea rickettsiosis in patients suffering from fever of unexplained origin [131,132,133,134,135]. For example, the prevalences of cat flea rickettsiosis among patients in Senegal and Kenya were 4.4% and 3.7%, respectively [136,137]. Furthermore, cat flea rickettsiosis and malaria share some similarities in their geographic distribution, seasonality, and clinical symptoms [6]. Studies were conducted among patients from Senegal (2075), Mali (100), Gabon (50), Tunisia (183), Algeria (266), Morocco (48), and France (400) from June 2010 to March 2012 [6]. Medical assessments were done, and blood samples were collected to evaluate and compare the epidemiology of *R. felis* and *Plasmodium* spp. by PCR (*R. felis* and *Plasmodium* spp.) and Giemsa staining (*Plasmodium* spp.). The attack rate of *R. felis* infection was highest among patients from Senegal (15%) compared to patients from the other countries (1–10%) [6]. Also, the attack rate was higher during the rainy season than the dry season. When compared to malaria, the attack rates of *R. felis* infection were also high in countries with high attack rates of malaria (Senegal, Gabon, and Mali) and vice versa (Algeria, Tunisia, Morocco, and France) [6]. Most of the countries with higher attack rates were in West Africa. In Nigeria, the rate of malaria infection is among the highest globally [138,139,140] but there is no information on the presence of *R. felis* infections. Based on the results from this study and the endemicity of malaria in Nigeria, it is very likely that *R. felis* infections also occur in Nigeria. The lack of recognition of this disease correlation in Nigeria indicates the need for its further investigation.

Tungiasis is an inflammatory skin disease caused by the flea *Tunga penetrans.* It occurs when the female parasite burrows into the skin. Initial burrowing by the gravid female is usually painless; symptoms, including itching and irritation, usually start to develop as the females become fully-developed into the engorged state [141]. Inflammation and ulceration may become severe, and multiple lesions in the feet can lead to difficulty in walking. Several published reports on tungiasis indicate a high prevalence of 30–50% of this condition in Nigeria [110,111,112,113]. In an early study on primary and post-primary school pupils in Rivers State (East Coastal region), 30.4% of 480 pupils suffered from tungiasis, more male pupils (33.8%) than female pupils (27.1%) were infested and a progressive decline in prevalence of tungiasis with age was observed [111]. A cross-sectional study was conducted among 615 students for the prevalence of tungasiasis in two government-run primary schools in Lagos state [127]. Tungiasis skin lesions were detected in 468 (76.1%) of the students [127]. School children infested with this disease often miss school due to the severe discomfort, from itching and pain. This flea needs further attention because *T. penetrans* was positive for *R. felis* DNA in a study in the Democratic Republic of Congo [142]; *T. penetrans* may thus serve as an unrecognized vector for the transmission of *R. felis* to humans in Nigeria.

## 4. Lice and Louse-Borne Diseases in Nigeria 

Contemporary information about the presence and prevalence of human pediculosis in Nigeria is mostly limited to public health reports focused on head louse pediculosis and its prevalence in pediatric populations (Table 3) [143,144,145,146,147,148,149].

Historically, the only well-described outbreak of clinical typhus occurred between 4 June and 15 September of 1945 in Jos, native town on the Bauchi Plateau in northern Nigeria, was the first typhus epidemic in West Africa [150,151]. Jos situated at a height of 4134 ft (~1260 m) and has a climate which is considerably cooler, especially at night, compared to elsewhere in Nigeria. This epidemic resulted in 126 cases, of which 32 (25%) died [150]. It was discovered that 20,000 of the population of Jos native town lived in overcrowded buildings, about half of the population did not possess a complete change of clothing, garment laundering and personal ablutions were limited, and almost everybody was infested with body lice, *Pediculus humanis corporis* [150]. Head lice, crab lice, and bed bugs were also found. Clinical symptoms of the patients were compatible with classical description of epidemic typhus, although the investigators emphasized rapid development of encephalitis, which dominated the clinical picture [152]. In a follow up laboratory study, isolates of *Rickettsia* sp. from body lice collected from the sick people or those living in the same compound during this outbreak were made by intraperitoneal inoculation of guinea pigs [151]. Ten *Rickettsia* sp. isolates were established from the blood of nine patients drawn on days 3–8 of acute illness (median = 5.5, mode = 5). All strains, whether isolated from patient’s blood or lice, produced a similar clinical picture in guinea pigs, including an incubation period between 5 and 10 days and temperature 40–40.6 °C (104–105 °F), death on of the day 2–3 of fever, and occasional development of scrotal reaction in male guinea pigs [151]. To complete investigation, serological testing was completed using the Weil-Felix test, rickettsial agglutination and complement fixation tests [151]. Accordingly, 82 out of 92 sera tested exhibited a high titer with OX19 (suggestive of a typhus group reaction) and 10/92 tested positive with OX2. Of 31 sera tested in the agglutination assay, 29 (93.5%) exhibited a higher titer with *R. prowazekii* antigen. The results of the complement fixation test corroborated these findings, thus implicating *R. prowazekii* as the etiological agent responsible for this deadly outbreak.

Later observations were made on patients with a diagnosis of a pyrexia of unknown origin in Enugu State (south central Nigeria); an infection with either or both scrub fever typhus and epidemic typhus was based on positive Weil-Felix reactions to *Proteus mirabilis* OXK and OX19 antigens on specimen sera from 211 patients examined [153]. Out of 211 samples examined, 154 sera tested positive with both *Proteus* OXK antigen and *Proteus* OX19 antigen, 57 sera tested positive with OXK antigen only. An outbreak of epidemic typhus is unlikely in the absence of body louse infestations; however, based on new discoveries of *Orientia* in Africa, a scrub typhus-like infection cannot be excluded [154,155].

The only published report of the occurrence of another louse-borne illness, relapsing fever due to *Borrelia recurrentis*, is known from Lagos State (western coastal region) [156]. Seven cases were identified and attributed to the return of Senegalese troops from the European war after the first outbreak was recorded in a British territory (Accra, Ghana) in 1922. Improved transport capabilities led to the introduction of the disease into Nigeria, along the Niger, through Delta State (central coastal region) to Lagos State. The current occurrence of *B. recurrentis* in Nigeria is not known but it should be considered as another health burden if body lice are present.

Head louse infestation is a common occurrence especially among children living in communities with poor hygienic practices. Multiple studies have reported the occurrence of head louse infestations in Nigeria [143,144,145,146,147,148,149] with most of them reporting a prevalence ranging from <1% to 45.6%. Also, there were significant differences in prevalence rates of infestation in rural (0–3% in Oyo State; one case detected in a school in Kwara State) and urban (4.2–6.9% in Oyo State; 0.1–3.1% in Kwara State) areas [143,145]. Some controversies on the role of the head louse as a vector for the transmission of diseases in humans persist. *Acinetobacter baumannii* has been detected by PCR in head lice from studies conducted in Thailand [157], Ethiopia [158], and Senegal [159] with prevalence of 1.45, 47, and 4% in the head lice respectively. No report on the detection of *A. baumannii* in head lice from Nigeria has been identified. However, one study detected multi-drug resistant (especially to imipenem) *A. baumannii* in clinical isolates in the University College Hospital, Oyo State [160]. Three out of five isolates analyzed from March to May 2015 were positive for molecular markers of resistance to imipenem [160]. Two other studies from Nigeria reported multidrug-resistant strains of *A. baumannii* from body fluids in patients admitted in tertiary hospitals [161,162]. Isolates with similar antibiotic-resistance profile and carrying *bla*TEM, *bla*CTX-M, and *bla*OXA genes have been identified from other global studies of *A. baumannii* [158,161,163,164,165]. Presently, there is no evidence of *A. baumannii* in head lice in Nigeria, but the role of lice as a vector of *A. baumannii* in Nigeria should be evaluated.

In a study conducted by Sangaré et al. [166], the presence of *B. quintana* was evaluated in nine African countries including Senegal and Mali, which are near neighbors of Nigeria. In total, 616 head lice and 424 body lice were tested for *B. quintana* DNA using PCR, and 2% of the head lice and 54% of the body lice tested positive. DNA of *B. quintana* was detected in two (0.52%) head lice tested in Senegal, indicating that it could be a possible vector for this pathogen. The occurrence of *B. quintana* in Nigeria is not proven yet; however, its presence is likely due to increased trade and migration activities among these countries. No other Nigerian studies were found on lice and louse-borne diseases.

## 5. Conclusions and Future Directions

The introductory section of this manuscript discussed the issues associated with the increasing impacts of non-mosquito vector-borne diseases on human populations, their known clinical and geographic overlap with mosquito-borne diseases, and the difficulties presented by other less-frequently recognized acute febrile illnesses caused by rickettsial pathogens in Africa in both humans and domestic animals. Our review and summary of retrieved literature has led us to the conclusion that Nigeria, as in many other countries in tropical and subtropical regions, is likely to have a broad spectrum of pathogens transmitted by various ectoparasites. However, the diseases they cause are generally overlooked due to the lack of public health and veterinary resources, the instability of the political environment, and the resulting widespread health crisis in the region [167]. We summarized here the scientific evidence available for the presence of ticks, fleas, and lice in Nigeria, the sine qua non for the vector-borne diseases they can transmit. However, precise information about the incidence and prevalence of specific disease transmitted by each of these vectors among sylvatic and domestic animals, and to people is very limited, and this is necessary to drive the flow of additional resources. Early investigations were focused on the veterinary economic impacts of these ectoparasites, while some recent publications using molecular methods give more accurate and specific data on the magnitude and complexity of the circulation of multiple non-mosquito vector-borne pathogens in Nigeria.

Because resources are limited, it is desirable to try to prioritize studies that may lead to the greatest impact. With respect to public health, the absence of reports related to human exposure to *R. typhi*, *R. africae*, and *R. felis* is puzzling; this appears to be similar to the situations in many other parts of the continent [6,131,137] and may be due to the erroneous perception that they are self-limiting and unlikely to be fatal. However, *R. africae* can have an impact on tourism as it is one of the most frequently-reported travel-associated infections; it causes less severe infections than *R. conorii*, which is also probably greatly underreported. Reports of louse-borne typhus are not known in Nigeria since the 1940s, suggesting existing adequate public health measures are in place coupled with the near extinction of circulating body lice as in many other parts of the globe. However, louse-borne typhus remains on the WHO epidemic threat list for Africa and the continuing political instability and health crisis in many African countries may be a classic background for new outbreaks in conflict areas, as previously happened in Burundi in 1995–1997 [168] and Rwanda in 2012 [169]. One other rickettsial disease, scrub typhus, has recently received more attention in the context of the diagnosis of scrub-typhus-like cases outside of its classic endemic area [170,171], evidence for human exposure during passive surveillance [154,172], and molecular detection of *Orientia* DNA in these areas [173,174], so it may have been overlooked in Nigeria as well. Furthermore, circulation of emerging pathogens such as *Candidatus* Neoehrlichia mikurenis [25], potential human pathogenicity of *E. ruminantium* [175], and other yet unidentified pathogens is likely to contribute to the list of febrile illnesses. Addressing these diseases contributes to several potential public health outcomes. Previous observations made in Sri Lanka have clearly demonstrated that including rickettsial diseases in the list of differential diagnoses improved patients’ outcomes, due to correct treatment leading to faster recovery and release from the hospital [176], and a significant reduction in hospitalisation costs for readily-treated diseases.

Morbidity and mortality of rickettsial diseases may also be masked by tick-borne haemorrhagic viral infections especially Crimean–Congo haemorrhagic fever (CCHF). CCHF virus is commonly transmitted by *H. rufipes* and *H. truncatum*, although other tick species have been implicated [177]. Continued exposure of the human population to CCHFV is well established in Nigeria [178]. Other zoonotic tick-borne viruses—including Jos virus, Dugbe virus, Bhanja virus, and Thogoto virus—may cause severe illness in humans but they are diagnosed infrequently [179]. The impact of these viruses on livestock animals is not fully understood despite frequent isolation of these viruses from ticks collected from livestock hosts [180].

Given the economic importance of cattle to Nigeria, it is even easier to make the argument that enhanced diagnosis and treatment of important domestic stock will pay for itself relatively quickly by reducing or eliminating the economic burden of stock diseases, especially tick-borne diseases. Nigeria is no stranger to the global trend toward urbanization, so improved treatment of companion animals will also reduce exposure of their owners to these endemic tropical diseases. Therefore, it is indeed important to conduct comprehensive epidemiological and ecological studies on human and veterinary vector-borne rickettsial diseases in Nigeria using the increasingly refined molecular tools now available. These studies must be linked to improvement in the training of public health, medical, and veterinary personnel to increase their awareness about these illnesses so they can provide adequate measures and approaches to their diagnosis, surveillance, control, and prevention. Furthermore, public education of physicians and other medical care providers should emphasize critical aspects of recognizing these illnesses which can be confused with malaria, chickungunya, and hemorrhagic fevers but are so much more treatable.

## Figures and Tables

**Table 1:** Summary of ticks and tick-borne pathogens in Nigeria.

Tick Species Collected	Vertebrate Host (Number)	Location	Infestation ^a^ (%)	Pathogen (%) ^b^		Detection Method	Study [Reference]
				Host Blood	Tick		
*Hyalomma impeltatum*	Camels (170)	Kano	59.9	*Rickettsia*	*Rickettsia*	PCR	Kamani et al. 2015 [29]
-	-	-	-	*aeschlimannii* (18.8)	*aeschlimannii* (4.2–50.0) ^c^	-	-
*Hyalomma rufipes*	-	-	20.3	-	*Rickettsia*	PCR	-
-	-	-	-	-	*aeschlimannii* (56.3–94.1) ^c^	-	-
*Hyalomma dromedarii*	-	-	18.8	-	*Rickettsia*	PCR	-
-	-	-	-	-	*aeschlimannii* (13.3) ^c^	-	-
*Hyalomma impressum*	-	-	1	-	ND	PCR	-
*Rhipicephalus sanguineus*	Dogs (181)	Plateau	NR	*Hepatozoon canis* (41.4)	*Ehrlichia canis* (23.7)	PCR	Kamani et al. 2013 [25]
*Haemaphysalis leachi*	-	Rivers	NR	*Ehrlichia canis* (12.7)	*Hepatozoon canis* (21.1)	PCR	-
*Rhipicephalus turanicus*	-	Kaduna	NR	*Rickettsia* spp. (8.8)	*Rickettsia* spp. (10.5)	PCR	-
-	-	Kwara	-	*Babesia rossi* (6.6)	*Candidatus* Neoehrlichia	-	-
-	-	-	-	-	mikurensis (5.3)	-	-
-	-	-	-	*Anaplasma platys* (6.6)	*Anaplasma platys* (1.9)	-	-
-	-	-	-	*Rickettsia conorii*	*Rickettsia conorii*	-	-
-	-	-	-	*israelensis* (NR)	*israelensis* (NR) ^d^	-	-
*Rhipicephalus sanguineus*	Dogs (100)	Plateau	73	*Babesia rossi* (53) ^e^	NT	PCR & RLB	Adamu et al. 2014 [28]
*Haemaphysalis leachi*	-	-	18	*Theileria* spp. (12.5) ^e^	NT	-	-
*Rhipicephalus turanicus*	-	-	2	*Ehrlichia canis* (6.9) ^e^	NT	-	-
-	-	-	-	*Anaplasma* spp. (6.9) ^e^	-	-	-
-	-	-	-	*Theileria equi* (4.2) ^e^	-	-	-
*Amblyomma variegatum*	Dogs (NR)	Plateau	70.2	NT	*Babesia* spp.(3.9) ^f^	PCR	Ogo et al. 2012 [24]
*Rhipicephalus*	Cattle (NR)	Nassarawa	20.6	NT	*Babesia bigemina* (1.3) ^f^	PCR	-
(*Boophilus*) *decoloratus*	-	-	-	-	-	-	-
*Rhipicephalus sanguineus*	-	-	9.2	-	*Babesia divergens* (0.6) ^f^	PCR	-
-	-	-	-	-	*Anaplasma marginale* (20.0) ^g^	-	-
-	-	-	-	-	*Rickettsia africae* (4.4–7.8) ^h^	-	-
*Rhipicephalus sanguineus*	*Rattus rattus* (48)	Plateau	46.5	*Bartonella* spp. (18.8)	*Bartonella* spp. (10.0) ^i^	PCR & Blood culture ^j^	Kamani et al. 2013 [26]
*Haemaphysalis leachi*	*R. norvegicus* (121)	-	3.5	*Bartonella* spp. (29.8)	ND	PCR & Blood culture ^j^	-
-	*Mus musculus* (6)	-	-	-	ND	PCR & Blood culture ^j^	-
-	*Cricetomys gambianus* (2)	-	-	-	*Bartonella* spp. (50.0)	PCR & Blood culture ^j^	-
*Hyalomma rufipes*	Cattle (NR)	Nigeria	34 ^k^	NT	*Babesia kinetes* (88)	Haemolymph	Dipeolu et al. 1984 [19]
-	-	-	-	-	-	smear stain	-
*Hyalomma truncatum*	-	-	32 ^k^	NT	*Babesia kinetes* (56)	Haemolymph	-
-	-	-	-	-	-	smear stain	-
*Hyalomma impressum*	-	-	19 ^k^	NT	*Babesia kinetes* (36)	Haemolymph	-
-	-	-	-	-	-	smear stain	-
*Hyalomma marginatum*	-	-	18 ^k^	NT	*Babesia kinetes* (72)	Haemolymph	-
-	-	-	-	-	-	smear stain	-
*Hyalomma impeltatum*	-	-	7 ^k^	NT	*Babesia kinetes* (43)	Haemolymph	-
-	-	-	-	-	-	smear stain	-
*Amblyomma variegatum*	Cattle (NR)	Plateau	141 ^k^	NT	*Rickettsia africae* (62)	PCR	Lorusso et al. 2013 [41]
*Rhipicephalus*	Cattle (120)	Nigeria	NR	*Babesia bigemina* (80)	NT	Blood smear stain	Akinboade et al. 1985 [17]
(*Boophilus*) spp.	-	-	-	-	-	-	-
*Rhipicephalus*	Dogs (400)	Borno	88	*Babesia canis* (12.0)	NT	Blood smear stain	Konto et al. 2014 [21]
(*Boophilus*) spp.	-	-	-	-	-	-	-
*Rhipicephalus sanguineus*	-	-	10.8	-	NT	-	-
*Hyalomma* spp.	-	-	0.9	-	NT	-	-
*Amblyomma variegatum*	-	-	0.3	-	NT	-	-
*Rhipicephalus*	Cattle (205)	Borno	63.4 ^l^	NT	NT	N/A	Musa et al. 2014 [42]
(*Boophilus*) *microplus*	-	-	-	-	-	-	-
*Amblyomma variegatum*	-	-	-	-	NT	-	-
*Hyalomma* spp.	-	-	-	-	NT	-	-
*Rhipicephalus sanguineus*	-	-	-	-	NT	-	-
*Ornithodorus* spp.	-	-	-	-	NT	-	-
*Amblyomma variegatum*	*Arvincanthis*	Delta	7.4	NT	NT	N/A	Ugbomoiko et al. 1991 [43]
-	*niloticus* (NR)	-	-	-	-	-	-
*Ixodes* sp.	*Lophuromys*	Edo	5.1	NT	NT	-	-
-	*sikapusi* (NR)	-	-	-	-	-	-
-	*Mastomys*	-	-	NT	-	-	-
-	*natalensis* (NR) ^m^	-	-	-	-	-	-
-	*Mus minutoides* (NR)	-	-	NT	-	-	-
-	*Leminiscomys*	-	-	NT	-	-	-
-	*striatus* (NR) ^m^	-	-	-	-	-	-
*Rhipicephalus*	Cattle (228)	Plateau	41.4	NT	NT	N/A	Lorusso et al. 2013 [27]
(*Boophilus*) *decoloratus*	-	-	-	-	-	-	-
*Rhipicephalus*	-	-	15.4	-	NT	-	-
(*Boophilus*) *annulatus*	-	-	-	-	-	-	-
*Rhipicephalus guilhoni*	-	-	12	-	NT	-	
*Rhipicephalus*	-	-	7.6	-	NT	-	-
(*Boophilus*) *geigyi*	-	-	-	-	-	-	-
*Hyalomma truncatum*	-	-	7.4	-	NT	-	-
*Amblyomma variegatum*	-		6.3	-	NT	-	-
*Rhipicephalus*	-	-	4.1	-	NT		-
(*Boophilus*) spp.	-	-	-	-	-	-	-
*Rhipicephalus*	-	-	4	-	NT	-	-
*simus group*	-	-	-	-	-	-	-
*Rhipicephalus turanicus*	-	-	1.2		NT	-	-
*Rhipicephalus sanguineus*	-	-	0.3	-	NT	-	-
*Hyalomma rufipes*	-	-	0.2	-	NT	-	-
*Rhipicephalus lunulatus*	-	-	<0.1	-	NT	-	-
*Rhipicephalus* spp.	Dogs (44)	Benue	34.1	NT	NT	N/A	Omudu et al. 2007 [44]
*Rhipicephalus*	-	-	29.5	NT	NT	-	-
(*Boophilus*) spp.	-	-	-	-	-	-	-
*Amblyomma* spp.	-	-	22.7	NT	NT	-	-
*Amblyomma* spp.	Goats (45)	Benue	20.1	NT	NT	N/A	Omudu et al. 2007 [44]
*Rhipicephalus* spp.	-	-	13.3	NT	NT	-	-
*Rhipicephalus*	-	-	11.1	NT	NT	-	-
(*Boophilus*) spp.	-	-	-	-	-	-	-
*Hyalomma* spp.	-	-	8.9	NT	NT	-	-
*Amblyomma* spp.	Cattle (43)	Benue	25.6	NT	NT	N/A	Omudu et al. 2007 [44]
*Hyalomma* spp.	-	-	18.6	NT	NT	-	-
*Rhipicephalus*	-	-	16.3	NT	NT	-	-
(*Boophilus*) spp.	-	-	-	-	-	-	-
*Rhipicephalus* spp.	-	-	9.3	NT	NT	-	-
*Amblyomma* spp.	Pigs (44)	Benue	20.5	NT	NT	N/A	Omudu et al. 2007 [44]
*Hyalomma* spp.	-	-	20.5	NT	NT	-	-
*Rhipicephalus*	-	-	15.9	NT	NT	-	-
(*Boophilus*) spp.	-	-	-	-	-	-	-
*Amblyomma* spp.	Sheep (45)	Benue	13.3	NT	NT	N/A	Omudu et al. 2007 [44]
*Rhipicephalus*	-	-	4.4	NT	NT	-	-
(*Boophilus*) spp.	-	-	-	-	-	-	-
*Hyalomma* spp.	-	-	8.9	NT	NT	-	-
*Rhipicephalus sanguineus*	Dogs (200)	Kogi	80	NT	NT	N/A	Abah et al. 2013 [45]
*Amblyomma variegatum*	Cattle (120)	Kaduna	22.5	NT	NT	N/A	Obadiah et al. 2012 [46]
*Rhipicephalus*	-	-	17.5	-	NT	-	-
(*Boophilus*) *decoloratus*	-	-	-	-	-	-	-
*Hyalomma* sp.	-	-	6.7	-	NT	-	-
*Rhipicephalus sanguineus*	-	-	3.3	-	NT	-	-
*Rhipicephalus sanguineus*	Dogs (202)	Ogun	89.6	NT	NT	N/A	Agbolade et al. 2008 [47]
*Haemaphysalis leachi*	-		78.7	-	NT	-	-
*Hyalomma rufipes*	Camels (1600)	Borno	34.9	NT	NT	N/A	James-Rugu et al. 2004 [48]
*Hyalomma dromedarii*	-	Yobe	30.4	-	NT	-	-
*Hyalomma truncatum*	-	-	10.6	-	NT	-	-
*Rhipicephalus*	-	-	24.2	-	NT	-	-
(*Boophilus*) *decoloratus*	-	-	-	-	-	-	-
*Amblyomma variegatum*	Dogs (230)	Borno	23.5	NT	NT	N/A	James-Rugu et al. 2004 [48]
*Rhipicephalus* spp.	-	Yobe	21.2	-	NT	-	-
*Rhipicephalus* (*Boophilus*)	-	-	40.8	-	NT	-	-
*decoloratus*	-	-	-	-	-	-	-
*Amblyomma lepidum*	-	-	7.1	-	NT	-	-
*Haemaphysalis leachi*	-	-	6.9	-	NT	-	-
*Amblyomma variegatum*	Cattle (2200)	Borno	11.4	NT	NT	N/A	James-Rugu et al. 2004 [48]
*Amblyomma lepidum*	-	Yobe	1.97	-	NT	-	-
*Hyalomma truncatum*	-	-	17.9	-	NT	-	-
*Hyalomma rufipes*	-	-	11.5	-	NT	-	-
*Haemaphysalis leachi*	-	-	8.8	-	NT	-	-
*Rhipicephalus evertsi*	-	-	17.8	-	NT	-	-
*Rhipicephalus* (*Boophilus*)	-	-	21.8	-	NT	-	-
*decoloratus*	-	-	-	-	-	-	-
*Rhipicephalus sanguineus*	-	-	8.9	-	NT	-	-
*Amblyomma variegatum*	Sheep (500)	Borno	50.0	NT	NT	N/A	James-Rugu et al. 2004 [48]
*Rhipicephalus* (*Boophilus*)	-	Yobe	50.0	-	NT	-	-
*decoloratus*	-	-	-	-	-	-	-
*Amblyomma variegatum*	Cattle (3150)	Borno	43.8	NT	NT	N/A	Opara et al. 2016 [49]
*Hyalomma* sp.	-	Yobe	24.7	-	NT	-	-
*Rhipicephalus* (*Boophilus*)	-	-	21.9	-	NT	-	-
*microplus*	-	-	-	-	-	-	-
*Dermacentor variabilis*	-	-	9.6	-	NT	-	-
*Amblyomma variegatum*	Cattle (1200)	Nasarawa	20.8	NT	NT	N/A	Tongjura et al. 2012 [50]
*Amblyomma lepidum*	-	-	14.3	-	NT	-	-
*Rhipicephalus* (*Boophilus*)	-	-	14.3	-	NT	-	-
*decoloratus*	-	-	-	-	-	-	-
*Rhipicephalus* (*Boophilus*)	-	-	12.8	-	NT	-	-
*annulatus*	-	-	-	-	-	-	-
*Hyalomma truncatum*	-	-	6.8	-	NT	-	-
*Amblyomma variegatum*	Sheep (1200)	Nasarawa	18.2	NT	NT	N/A	Tongjura et al. 2012 [50]
*Amblyomma lepidum*	-	-	8.6	-	NT	-	-
*Hyalomma truncatum*	-	-	5.6	-	NT	-	-
*Rhipicephalus* (*Boophilus*)	-	-	10.0	-	NT	-	-
*decoloratus*	-	-	-	-	-	-	-
*Rhipicephalus* (*Boophilus*)	-	-	7.6	-	NT	-	-
*annulatus*	-	-	-	-	-	-	-
*Amblyomma variegatum*	Goats (1200)	Nasarawa	15.5	NT	NT	N/A	Tongjura et al. 2012 [50]
*Rhipicephalus* (*Boophilus*)	-	-	7.1	-	NT	-	-
*decoloratus*	-	-	-	-	-	-	-
*Amblyomma lepidum*	-	-	6.6	-	NT	-	-
*Rhipicephalus* (*Boophilus*)	-	-	6.4	-	NT	-	-
*annulatus*	-	-	-	-	-	-	-
*Hyalomma truncatum*	-	-	3.7	-	NT	-	-
*Rhipicephalus evertsi*	Cattle (317)	Oyo	37.6	NT	NT	N/A	Ameen et al. 2014 [51]
*evertsi*	-	-	-	-	-	-	-
*Rhipicephalus* (*Boophilus*)	-	-	22.1	-	NT	-	-
*decoloratus*	-	-	-	-	-	-	-
*Rhipicephalus* (*Boophilus*)	-	-	17.8	-	NT	-	-
*annulatus*	-	-	-	-	-	-	-
*Amblyomma variegatum*	-	-	15.8	-	NT	-	-
*Rhipicephalus*	-	-	10.7	-	NT	-	-
*appendiculatus*	-	-	-	-	-	-	-
*Haemaphysalis leachi*	-	-	5.7	-	NT	-	-
*Rhipicephalus evertsi*	Goats (210)	Oyo	33.3	NT	NT	N/A	Ameen et al. 2014 [51]
*evertsi*	-	-	-	-	-	-	-
*Rhipicephalus* (*Boophilus*)	-	-	24.8	-	NT	-	-
*decoloratus*	-	-	-	-	-	-	-
*Rhipicephalus* (*Boophilus*)	-	-	18.1	-	NT	-	-
*annulatus*	-	-	-	-	-	-	-
*Amblyomma variegatum*	-	-	11.9	-	NT	-	-
*Rhipicephalus*	-	-	6.2	-	NT	-	-
*appendiculatus*	-	-	-	-	-	-	-
*Haemaphysalis leachi*	-	-	2.9	-	NT	-	-
*Rhipicephalus evertsi*	Sheep (104)	Oyo	28.8	NT	NT	N/A	Ameen et al. 2014 [51]
*evertsi*	-	-	-	-	-	-	-
*Rhipicephalus* (*Boophilus*)	-	-	18.3	-	NT	-	-
*decoloratus*	-	-	-	-	-	-	-
*Rhipicephalus* (*Boophilus*)	-	-	11.5	-	NT	-	-
*annulatus*	-	-	-	-	-	-	-
*Amblyomma variegatum*	-	-	9.6	-	NT	-	-
*Rhipicephalus*		-	8.7	-	NT	-	-
*appendiculatus*	-	-	-	-	-	-	-
*Haemaphysalis leachi*	-	-	4.8	-	NT	-	-
*Rhipicephalus sanguineus*	Dogs (820)	Edo	19.5	NT	NT	N/A	Ugochukwu et al. 1985 [52]
-	-	Delta	-	-	-	-	-
*Rhipicephalus sanguineus*	Dogs (396)	Kwara	19.2	NT	NT	N/A	Ugbomoiko et al. 2008 [53]
*Ixodes* sp.	-	-	4.5	-	NT	-	-
*Rhipicephalus sanguineus*	Dogs (130)	Benue	80.5	NT	NT	N/A	Amuta et al. 2010 [54]
*Rhipicephalus* (*Boophilus*)	-	-	14.6	-	NT	-	-
*annulatus*	-	-	-	-	-	-	-
*Hyalomma truncatum*	-	-	4.9	-	NT	-	-
*Rhipicephalus sanguineus*	Dogs (157)	Edo	53.6	NT	NT	N/A	Isaac et al. 2016 [55]
*Rhipicephalus pulchellus*	-	-	42.3	-	NT	-	-
*Rhipicephalus*	-	-	7.4	-	NT	-	-
(*Boophilus*) *decoloratus*	-	-	-	-	-	-	-
*Hyalomma truncatum*	Cattle (450)	Anambra	34.8	NT	NT	N/A	Ikpeze et al. 2011 [56]
*Rhipicephalus*	-	Enugu	23.1	-	NT	-	-
*appendiculatus*	-	-	-	-	-	-	-
*Rhipicephalus*	-	-	22.1	-	NT	-	-
(*Boophilus*) *annulatus*	-	-	-	-	-	-	-
*Amblyomma variegatum*	-	-	20.1	-	NT	-	-
*Rhipicephalus sanguineus*	Humans (11)	Imo	NR	NT	NT	N/A	Okoli et al. 2006 [57]
-	-	Dogs (2)	-	-	NT	-	-
-	-	Sheep (3)	-	-	NT	-	-
*Rhipicephalus sanguineus*	Dogs (150)	Cross River	NR	NT	NT	N/A	Arong et al. 2013 [58]
*Haemaphysalis leachi*	-	-	-	-	NT	-	-
*Rhipicephalus*	-	-	-	-	NT	-	-
(*Boophilus*) *decoloratus*	-	-	-	-	-	-	-
*Rhipicephalus*	Goats (NR)	Enugu	NR	NT	NT	N/A	Ugochukwu et al. 1985 [59]
(*Boophilus*) *decoloratus*	-	-	-	-	-	-	-
*Amblyomma variegatum*	-	-	NR	-	NT	-	-
*Amblyomma variegatum*	Cattle (NR)	Kano	NR	NT	NT	N/A	Unsworth K. 1952 [60]
*Amblyomma splendidum*	-	Katsina	NR	-	NT	-	-
*Rhipicephalus*	-	Plateau	NR	-	NT	-	-
(*Boophilus*) *decoloratus*	-	-	-	-	-	-	-
*Hyalomma* spp.	-	Niger	NR	-	NT	-	-
*Rhipicephalus* spp.	-	Sokoto	NR	-	NT	-	-
-	-	Adamawa	-	-	-	-	-
-	-	Bauchi	-	-	-	-	-
-	-	Borno	-	-	-	-	-
-	-	Kwara	-	-	-	-	-
NR	Cattle (120)	Borno	N/A	*Anaplasma* spp. (5.8)	N/A	Blood smear stain	Paul et al. 2016 [22]
-	-	-	-	*Babesia* spp. (4.2)	-	-	-
NR	Goats (72)	Nassarawa	N/A	*Anaplasma* spp. (15.3)	N/A	Blood smear stain	Opara et al. 2016 [23]
-	-	-	-	*Babesia* spp. (5.6)	-	-	-
NR	Sheep (32)	Nassarawa	N/A	*Anaplasma* spp. (9.4)	N/A	Blood smear stain	Opara et al. 2016 [23]
-	-	-	-	*Babesia* spp. (3.1)	-	-	-
NR	Dogs (101)	Abuja	N/A	*Babesia canis* (8.9)	N/A	Blood smear stain	Jegede et al. 2014 [20]
NR	Dogs (120)	Abuja	N/A	*Babesia* spp.	N/A	Blood smear stain	Obeta et al. 2009 [61]
NR	Dogs (100)	Plateau	N/A	*Ehrlichia canis* (11)	N/A	PCR	Kamani et al. 2013 [62]
NR	Cattle (704)	Plateau	N/A	*Theileria mutans* (61.8)	N/A	PCR, RLB	Lorusso et al. 2016 [63]
-	-	-	-	*Theileria velifera* (49.4)	-	-	-
-	-	-	-	*Anaplasma marginale* (38.1)	-	-	-
-	-	-	-	*Theileria taurotragi* (36.9)	-	-	-
-	-	-	-	*Anaplasma* sp. (*Omatjenne*) (33.9)	-	-	-
-	-	-	-	*Anaplasma centrale* (8.1)	-	-	-
-	-	-	-	*Babesia bigemina* (8.1)	-	-	-
-	-	-	-	*Rickettsia* spp. (2.7)	-	-	-
-	-	-	-	*Babesia bovis* (2.3)	-	-	-
-	-	-	-	*Erhlichia ruminantium* (1.1)	-	-	-
-	-	-	-	*Anaplasma platys* (3.8)	-	-	-
NR	Cattle (100)	Oyo	N/A	*Babesia bigemina* (9, 93.0) ^n^	N/A	Blood smear stain	Akinboade et al. 1984 [18]
-	-	-	-	-	-	IFA	-
-	-	-	-	*Anaplasma marginale* (8.9, 68.0) ^n^	-	Blood smear stain	-
-	-	-	-	-	-	IFA	-
-	-	-	-	*Babesia bovis* (3.3, 54.5) ^n^	-	Blood smear stain	-
-	-	-	-	-	-	IFA	-
-	-	-	-	*Anaplasma centrale* (0.75)	-	Blood smear stain	-
-	-	-	-	*Eperythrozoon* (0.75)	-	Blood smear stain	-
-	-	-	-	*Theleria* spp. (0.75)	-	Blood smear stain	-
NR	Cattle (500)	Nigeria	N/A	*Anaplasma marginale*	N/A	IFA, CT, CA	Ajayi et al. 1986 [64]
-	-	-	-	(79.4, 40.0, 25.0)	-	-	-
-	-	-	-	*Babesia bigemina* (29.4)	-	IFA	-
-	-	-	-	*Babesia bovis* (14.1)	-	IFA	-

NT: Not tested, NR: Not recorded, N/A: Not applicable, ND: Not detected, IFA: Indirect fluorescent antibody test, CT: Card agglutination, CA: Capillary tube-agglutination, PCR: Polymerase chain reaction, RLB: Reverse line blotting. ^a^ Level of infestation (%) is compared to the total pool of ticks or vertebrate host examined in the study otherwise where stated. ^b^ Level of infection (%) is compared to the total ticks or hosts tested in the study otherwise where stated. ^c^ Prevalence of SFG *Rickettsia* detected in pools of ticks using different set of tick primers and PCR methods. ^d^ Pathogen was detected only in one pool of *Rh. sanguineus* collected. ^e^ Prevalence is based on the number of dogs positive for at least one pathogen. ^f^ Pathogens were detected only in pools of *A. variegatum.* ^g^ Pathogens were detected only in pools of *Rh. decoloratus.* ^h^ *R. africae* was detected in all pools of ticks with varying prevalence. ^i^ Prevalence is based on pools of *Rh. sanguineus* tested. ^j^ *Bartonella* sp. was cultured from only 30 rodent blood samples. ^k^ Number of each species of tick detached from cattle. ^l^ Prevalence of infestation was for all tick pools collected in the study. ^m^ *Ixodes* sp. was not collected from the hosts. ^n^ Prevalence of *B. bigemina*, *A. marginale*, and *B. bovis* from blood smear stain and IFA respectively. ^o^ Prevalence of *A. marginale* from IFA, CT, and CA respectively.

**Table 2:** Summary of fleas and their associated pathogens in Nigeria.

Species Collected	Vertebrate Host (Number)	Location	Infestation (%)	Pathogen (%)		Detection Method	Study [Reference]
				Host Blood	Fleas		
*Xenopsylla cheopsis*	*Rattus rattus* (48)	Plateau	NR	*Bartonella* spp. (18.8)	*Bartonella* spp. (50) ^a^	PCR, Blood culture ^b^	Kamani et al. 2013 [26]
*Ctenophthalmus* sp.	*R. norvegicus* (121)	-	NR	*Bartonella* spp. (29.8)	*Bartonella* spp. (66.7) ^c^	PCR, Blood culture ^b^	-
-	*Mus musculus* (6)	-	-	ND	-	PCR, Blood culture ^b^	-
-	*Cricetomys gambianus* (2)	-	-	*Bartonella* spp. (50.0)	-	PCR, Blood culture ^b^	-
*Ctenocephalides canis*	Goats (2)	Oyo	NR	*Babesia motasi*	NT	NR	Opasina. 1983 [126]
*Ctenocephalides canis*	Dogs (396)	Kwara	32.1	NT	NT	N/A	Ugbomoiko et al. 2008 [53]
*Pulex irritans*	-	-	6.6	NT	-	-	-
*Tunga penetrans*	-	-	0.5	NT	-	-	-
*Ctenocephalides* sp.	Dogs (44)	Benue	38.6	NT	NT	N/A	Omudu et al. 2007 [44]
*Pulex* sp.	-	-	6.8	NT	NT	-	-
*Tunga* sp.	Pigs (44)	Benue	77.3	NT	NT	N/A	Omudu et al. 2007 [44]
*Tunga* sp.	Goats (45)	Benue	22.2	NT	NT	N/A	Omudu et al. 2007 [44]
*Tunga* sp.	Sheep (45)	Benue	26.7	NT	NT	N/A	Omudu et al. 2007 [44]
*Tunga penetrans*	Humans (5595)	Lagos	49.5	NT	NT	N/A	Ejezie G. 1981 [110]
*Tunga penetrans*	Humans (480)	Rivers	30.4	NT	NT	N/A	Arene, F. O. I. 1984 [111]
*Xenopsylla cheopsis*	*Arvincanthis*	Delta	22.4	NT	NT	N/A	Ugbomoiko et al. 1991 [43]
-	*niloticus* (NR)	-	-	-	-	-	-
*Xenopsylla braziliensis*	*Lophuromys*	Edo	5.1	NT	NT	-	-
-	*sikapusi* (NR) ^d^		-	-	-	-	-
-	*Mastomys*	-	-	NT	-	-	-
-	*natalensis* (NR)	-	-	-	-	-	-
-	*Mus minutoides* (NR) ^d^	-	-	NT	-	-	-
-	*Leminiscomys*	-	-	NT	-	-	-
-	*striatus* (NR) ^d^	-	-	-	-	-	-
-	*Rattus rattus* (NR)	-	-	NT	-	-	-
*Tunga penetrans*	Humans (547)	Lagos	45.2	NT	NT	N/A	Ugbomoiko et al. 2007 [113]
*Tunga penetrans*	Humans (545)	Lagos	22.4	NT	NT	N/A	Ugbomoiko et al. 2017 [127]
*Ctenocephalides canis*	Dogs (202)	Ogun	13.4	NT	NT	N/A	Agbolade et al. 2008 [47]
*Xenopsylla cheopsis*	*Rattus rattus* (50)	Osun	36	NT	NT	N/A	Ogunniyi et al. 2014 [117]
*Ctenocephalides canis*	Dogs (338)	Anambra	26.3	NT	NT	N/A	Chukwu et al. 1985 [114]
*Echidnophaga gallinacean*	-	-	2.1	-	NT	-	-
*Ctenocephalides canis*	Dogs (820)	Edo	NR	NT	NT	N/A	Ugochukwu et al. 1985 [52]
-	-	Delta	-	-	-	-	-
*Ctenocephalides canis*	Humans (NR)	Oyo	NR	NT	NT	N/A	Fagbemi et al. 1981 [109]
*Ctenocephalides felis*	Cattle (NR)	NR	NR	NT	NT	N/A	Shaibu et al. 2011 [128]

NT: Not tested; NR: Not recorded; N/A: Not applicable; PCR: Polymerase chain reaction. ^a^ Prevalence of *Bartonella* spp. in pools of *X. cheopsis.* ^b^ *Bartonella* sp. was cultured from only 30 rodent blood samples. ^c^ Prevalence of *Bartonella* spp. in pools of *Ctenophthalmus* sp. ^d^ Only *X. cheopsis* was collected on the vertebrate host.

**Table 3:** Summary of lice and their associated pathogens in Nigeria.

Species Collected	Vertebrate Host (Number)	Location	Infestation (%)	Pathogen (%)		Detection Method	Study [Reference]
				Host Blood	Lice		
*Pediculus humanus corporis*	Humans (126)	Plateau	NR	*Rickettsia prowazekii* (100) ^a^	NT	Weil-Felix	Montgomery et al. 1947 [152]
*Pediculus humanus capitis*	Humans (2333)	Oyo	4.8 ^b^	NT	NT	N/A	Ogunrinade et al. 1984 [143]
*Pediculus humanus capitis*	Humans (2704)	Osun	12.7 ^b^	NT	NT	N/A	Jinadu, M. K. 1985 [144]
*Pediculus humanus capitis*	Humans (7360)	Abia, Imo	5.7 ^b^	NT	NT	N/A	Arene et al. 1985 [147]
-	-	Akwa Ibom	-	-	-	-	-
-	-	Bayelsa, Delta	-	-	-	-	-
-	-	Cross River	-	-	-	-	-
-	-	Edo, Ondo	-	-	-	-	-
-	-	Rivers	-	-	-	-	-
*Pediculus humanus capitis*	Humans (6882)	Kwara	3.7 ^b^	NT	NT	N/A	Ebomoyi, E. 1994 [146]
*Pediculus humanus capitis*	Humans (2898)	Kwara	2.0 ^b^	NT	NT	N/A	Ebomoyi, E. 1988 [145]
*Pediculus humanus capitis*	Humans (1000)	Kwara	8.8 ^b^	NT	NT	N/A	Okwa et al. 2010 [149]
*Pediculus humanus capitis*	Humans (726)	Rivers	45.6 ^b^	NT	NT	N/A	Gboeloh et al. 2013 [148]
NR	Humans (NR)	Enugu	N/A	*Rickettsia prowazekii*	N/A	Weil-Felix	Emejuaiwe et al. 1978 [153]

NT: Not tested, NR: Not recorded, N/A: Not applicable. ^a^ Testing was performed on hospitalized patients. ^b^ Overall prevalence of infestation in the study.
